# Experimental Study on the Chlorine-Induced Corrosion and Blister Formation of Steel Pipes Coated with Modified Polyethylene Powder

**DOI:** 10.3390/polym16172415

**Published:** 2024-08-26

**Authors:** Myung Kue Lee, Dongchan Kim, Min Ook Kim

**Affiliations:** 1 Department of Civil and Environmental Engineering, Jeonju University, 303 Cheonjam-ro, Wansan-gu, Jeonju 55069, Jeollabuk-do, Republic of Korea; concrete@jj.ac.kr; 2 Department of Civil Engineering, Seoul National University of Science and Technology, 232 Gongneung-ro, Nowon-gu, Seoul 01811, Republic of Korea; kdc0357@seoultech.ac.kr

**Keywords:** steel pipe, modified polyethylene powder, surface coating, corrosion resistance

## Abstract

In this study, chlorine-induced corrosion and blister formation on steel pipes (SPs) coated with modified polyethylene powder (MPP) were evaluated through various tests, including chlorine exposure, wet immersion, and temperature gradient experiments. The results confirmed that the extent of corrosion and iron leaching varied with the coating type as expected. In batch leaching tests, no corrosion was observed on modified polyethylene-coated steel pipes (MPCSPs) within a chlorine concentration range of 0 mg/L to 10 mg/L; similarly, there were no significant changes in specimen weight or iron levels. In contrast, the control group with uncoated SPs exhibited significant iron leaching and corrosion, a trend consistent in sequential leaching experiments. SEM analysis after a month of chlorine exposure revealed no significant corrosion on MPCSPs, and SEM-EDX confirmed no major changes in the carbon bond structure, indicating resistance to high chlorine concentrations. Comparative analysis of wet immersion and temperature gradient tests between MPCSP and conventional epoxy-coated SP (ECSP) specimens revealed that MPCSPs did not develop blisters even after 100 days of immersion, whereas ECSPs began showing blisters as early as 50 days. In temperature gradient tests, MPCSPs showed no blisters after 100 days, while ECSPs exhibited severe internal coating layer blisters.

## 1. Introduction

Steel pipes (SPs) are fundamental components of modern infrastructure, extensively used in transporting fluids across various industries, including oil and gas, water treatment, and chemical processing [1,2,3]. Despite their widespread utility, the longevity and performance of SPs are compromised by corrosion [4,5,6]. The economic implications of corrosion are substantial, requiring significant annual expenditures on the maintenance, repair, and replacement of infrastructure globally. To prevent corrosion, protective coatings are applied to SPs, enhancing their resistance to environmental and chemical aggressors. Among the variety of coatings available, polyethylene-based coatings have gained prominence due to their effective barrier properties, mechanical strength, and cost-efficiency [7,8,9]. Recent studies have significantly advanced our understanding of various aspects of pipeline corrosion, providing critical insights into prevention strategies, mitigation techniques, and the fundamental mechanisms driving corrosion processes [10,11,12,13,14,15,16]. However, the dynamic and aggressive conditions to which pipes are exposed—ranging from high temperatures and pressures to chemically active environments—demand continuous improvements in coating formulations to enhance protective capabilities.

In the Republic of Korea, most water treatment facilities use chlorine as a disinfectant, which can cause corrosion in pipes [17,18]. Moisture in contact with pipes acts as an electrolyte in electrochemical reactions, and the dissolution of acidic substances such as H_2_S and CO_2_ in water can accelerate the corrosion of SPs [19]. Previous research has shown that the durability of surface-coated SPs is greatly affected by the moisture penetration resistance of the coating material and environmental conditions such as temperature and humidity [20]. Additionally, exposure to high temperatures can significantly reduce the effective lifespan of the coating [21,22,23]. As a countermeasure, the interiors of SPs are coated with materials such as epoxy, urethane, and urea [3,24,25]. However, over time, the chlorine in tap water can deform these interior coatings, leading to internal corrosion. Therefore, the development of effective coating materials that can prevent pipe corrosion caused by chlorine, moisture penetration, exposure to temperature, and humidity is significant.

In the case of underground coated SPs, applied coatings should last at least 20 years to ensure the suitability of use and stability of the pipe system [26]. Surface coatings correctly applied are expected to protect the workability and fittings of SPs over the long term and effectively prevent corrosion [27]. Accordingly, polymeric coatings have been widely accepted to protect onshore and offshore pipelines [28]. Specifically, three-layer polypropylene coating (TLPC) and epoxy have been applied to SPs exposed to corrosive environments [29]. TLPC consists of a thin epoxy resin layer, a modified middle layer of polypropylene, and an external polypropylene layer. They demonstrate excellent adhesion, effectively isolating the pipe from harmful substances and preventing corrosion. Despite the effectiveness of TLPCs in preventing corrosion on SPs, further experimental study is necessary for several reasons. Firstly, improved coatings can significantly boost the lifespan and reliability of SPs by providing better corrosion resistance and mechanical wear properties. This not only helps by reducing maintenance costs but also minimizes the environmental risks associated with leaks and failures. Additionally, the development of innovative, cost-effective coatings can help industries meet increasingly stringent safety and environmental regulations. Furthermore, advancements in smart coating technologies, which integrate sensors to monitor the health and integrity of SPs, represent a forward leap in predictive maintenance strategies. This can lead to more timely interventions, preventing failures before they occur and ensuring safer operations. Consequently, continued research and development in this area is essential for enhancing the functionality and durability of SPs, making them more sustainable and efficient in their applications across various sectors.

In this study, the chlorine-induced corrosion of modified polyethylene-coated steel pipes (MPCSPs), which underwent extensive surface pretreatment including degreasing, acid treatment, phosphate coating, and heating, was investigated experimentally. The microstructural integrity of the coating surface was also examined to determine the extent of corrosion. Additionally, the occurrence of surface blisters on coated pipe specimens exposed to various conditions of temperature, moisture, and exposure duration was assessed through visual inspection. This research aims to develop an MPCSP variant with enhanced corrosion resistance and to evaluate its performance compared to conventional epoxy coatings.

## 2. Research Significance

This study explores the potential of modified polyethylene powder (MPP) as a coating material that offers enhanced corrosion resistance compared to traditional coatings. This is vital in industries such as chemical processing and offshore oil drilling, where pipe durability is crucial for safety and operational efficiency. The research aims to provide empirical data on the effectiveness, durability, and commercial viability of MPP coatings. The results could lead to more reliable and durable pipe systems, reducing maintenance frequency and costs. Furthermore, this research could contribute to the development of coatings that are not only more effective but also environmentally friendly. This study is part of an ongoing research project on smart pipe systems equipped with sensors for improved maintenance strategies. Additional test results from MPCSPs exposed to harsh marine environments will be reported.

## 3. Experimental Design

**Figure 1:**
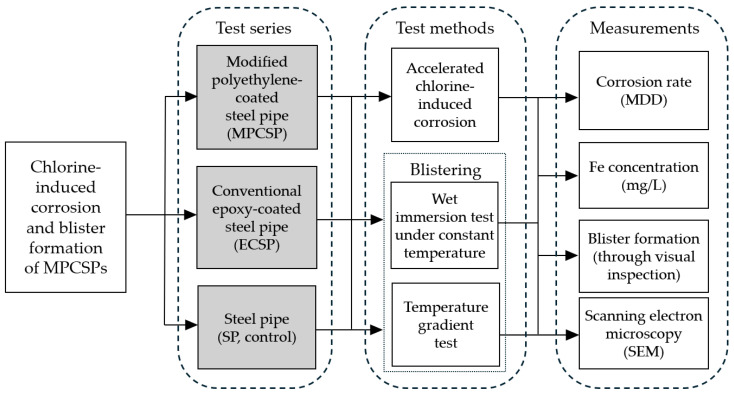
Test series, methods, and measurements considered in this study.

An experimental campaign was designed to investigate the effect of chlorine on the corrosion of coated and uncoated SP samples. Surface inspection was also performed to check if blisters formed on the specimens after the wet immersion and thermal gradient experiments were completed. The details of the prepared test series, testing methods, and measurements are shown in Figure 1. It should be noted that the coating in Figure 1 refers to the internal coating, and the exterior has been coated entirely with TLPC. As mentioned previously, a conventional epoxy-coated steel pipe (ECSP) was chosen for the comparison purpose. The selection of MPP, phosphate film, epoxy primer, modified PE adhesive layer, and resin layer in this study was carefully made to maximize corrosion protection and adhesion, with consideration for field application. MPP provides an initial barrier against corrosion, while the phosphate film enhances adhesion and offers additional corrosion resistance. The epoxy primer ensures strong bonding and adds resistance to moisture and chemicals. The modified PE adhesive layer further strengthens the adhesion between layers, and the resin layer adds a smooth, durable, and environmentally resistant finish. Together, these materials create a robust, long-lasting protective piping system.

### 3.1. Test Specimens and Coating Processes

**Figure 2:**
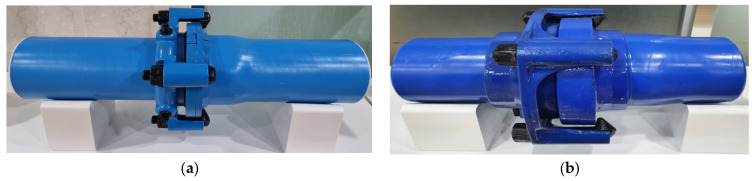
Representative specimens prepared through pretreatment process: (**a**) modified polyethylene-coated steel pipe (MPCSP); (**b**) epoxy-coated steel pipe (ECSP).

Figure 2 displays the prepared MPCSP and ECSP specimens after undergoing several coating processes. MPP was applied inside the MPCSP specimens, while the exterior underwent treatment with a phosphate film. This was followed by an extrusion coating of an epoxy primer, a modified PE adhesive layer, and a resin layer. A corrosion experiment on conventional SPs was also conducted for comparison purposes.

**Figure 3:**
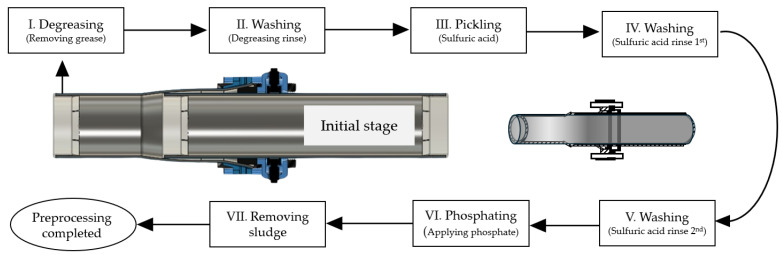
Chemical pretreatment process applied in steel pipes investigated in this study.

A chemical pretreatment process was applied to prevent the formation of an oxidation film layer and delamination (refer to Figure 3). The chemical pretreatment involves a series of reactions where Zn(H_2_PO_4_)_2_ decomposes into ZnHPO_4_ and H_3_PO_4_. Additionally, 3ZnHPO_4_ decomposes into Zn_3_(PO_4_)_2_ and H_3_PO_4_. Furthermore, 2H_3_PO_4_ reacts with Fe, producing Fe(H_2_PO_4_)_2_, H_2_, and the precipitate FePO_4_. Ultimately, Fe(H_2_PO_4_)_2_ reacts with 2Zn(H_2_PO_4_)_2_ to decompose into the main component of the phosphophyllite coating, Zn_2_Fe(PO_4_)_2_, and 4H_3_PO_4_.

After completing the chemical pretreatment, both the interior and exterior of the specimens were coated using a three-layer method. The interior coating involved heating the pipe to a predetermined temperature after pretreatment and applying a powder lining to thermally fuse modified PE powder, resulting in a corrosion-resistant coating consisting of an epoxy primer and modified PE powder lining. The exterior was coated by first applying an epoxy primer, followed by the co-extrusion of modified PE and high-density PE onto steel pipes that had undergone special pretreatment and heating. Thus, the exterior coating comprises an epoxy primer, modified PE, and high-density PE applied through extrusion.

### 3.2. Chlorine-Induced Corrosion Test

**Figure 4:**
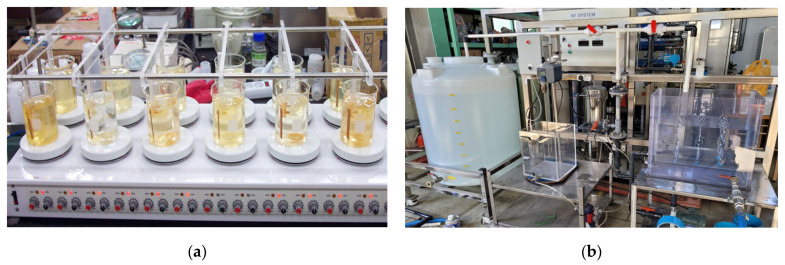
Chloride-induced accelerated corrosion test setup: (**a**) Batch leaching; (**b**) Sequential leaching.

Batch and sequential leaching experiments were conducted on MPCSP and SP specimens to evaluate the corrosion rate when exposed to chlorine. The setup for the batch leaching experiment, depicted in Figure 4a, includes a temperature control device, a magnetic stirring bar, a stirring device, and a hot plate. Rectangular specimens (30 mm × 20 mm) were prepared with a 5.0 mm hole at the top to facilitate suspension inside the experimental apparatus. Each specimen was secured in the batch leaching experiment device and 1.0 L of tap water was added to a beaker. A 12% NaOCl solution was diluted with ultrapure distilled water to achieve a concentration of 1000 mg/L.

In this study, the chlorine injection concentration ranged from 0 to 10 mg/L (Cl_2_), with corrosion and iron leaching levels measured over a period of seven days. To determine the appropriate concentration levels and time intervals for the main experiment, a series of pre-tests were conducted. These pre-tests involved varying chlorine concentrations and monitoring their effects on corrosion and iron leaching over different time periods. The results from these preliminary tests allowed us to identify the specific concentration levels and time intervals that would provide the most accurate and reliable data for the main study. By analyzing the trends observed during the pre-tests, we ensured that the chosen parameters were sufficient to capture the onset and progression of corrosion and iron leaching, while also preventing any significant experimental errors or inconsistencies

The sequential leaching experiment, illustrated in Figure 4b, utilized specimens of the same size of 30 mm × 20 mm. Unlike the batch experiments, tap water in the sequential experiment was cycled with a 12% NaOCl solution diluted to 1000 mg/L, injecting a chlorine concentration of 10 mg/L to simulate extreme conditions. Corrosion and iron leaching concentrations were measured every five days over 30 days to assess corrosion resistance. The specimens were suspended in the sequential experiment device as shown in Figure 4b.

### 3.3. Blistering Test

Wet immersion testing at a constant temperature was performed to assess whether blisters formed on the coated specimens and to evaluate their permeability. This test aimed to identify the point at which blisters occur under constant water exposure at a specified temperature. MPCSP (150A, 600A) and ECSP (150A) specimens were immersed in a small water tank within an oven maintained at 68 °C.

**Figure 5:**
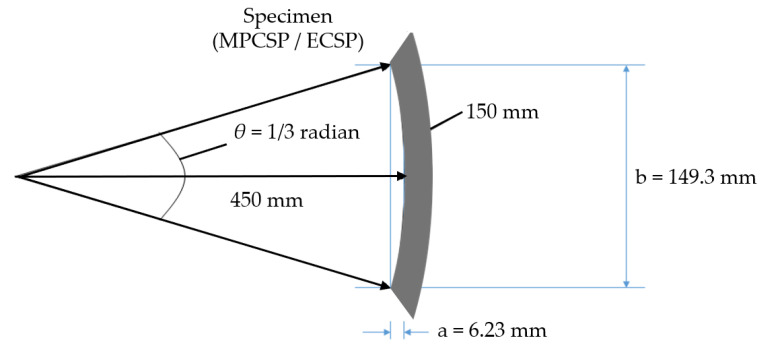
Specimen (900A) details (for blistering test) to reflect the curvature.

A thermal gradient experiment was also conducted to examine the permeability and potential blister formation. Specimens of the same type and size were exposed in water tanks at varying temperatures. The experimental setup was specially designed to accommodate the curvature of the specimens, which affects their shape when cut from coated pipe products, as depicted in Figure 5 and Table 1.

**Figure 6:**
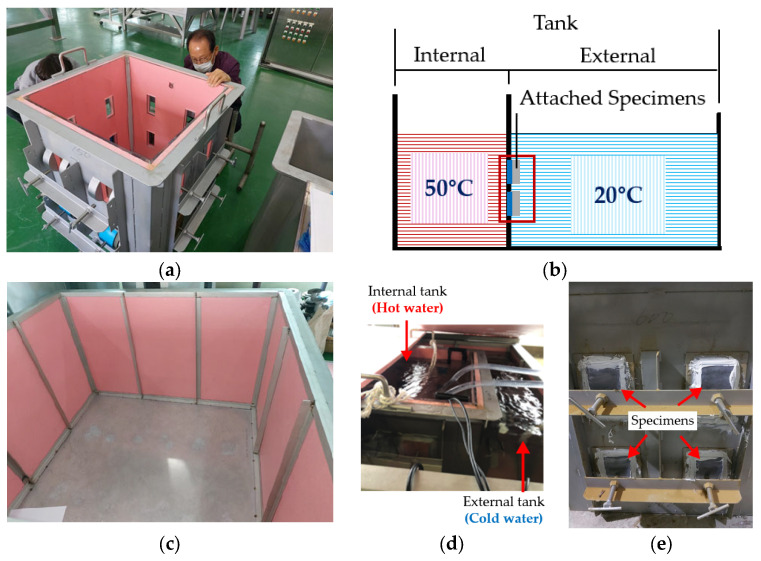
Thermal gradient test: (**a**) internal tank; (**b**) schematic representation of test; (**c**) external tank; (**d**) photo of the test in progress; (**e**) test specimens attached between two tanks.

As illustrated in Figure 6a,b, the setup included inner and outer tanks; the inner tank contained hot water at 50 °C, and the outer tank held cold water at 20 °C. The specimens were attached to the outer wall of the inner tank, as shown in Figure 6c. This arrangement exposed the outer steel portion to the 20 °C water and the inner coated portion to the 50 °C water, creating conditions conducive to water penetration. The presence and extent of blisters were visually inspected during the experiments.

### 3.4. Measurements and Surface Analysis

The corrosion rate, expressed as MDD (milligrams per decimeter squared per day), was estimated based on changes in the measured weights of the specimens before and after the experiment. The weights were measured after all corrosion products and scale had been removed, and the specimens had been thoroughly dried. *MDD* can be determined using Equation (1) as follows:

(1) $$MDD=\frac{{Weight}_{before}\left(mg\right)-{Weight}_{after}\left(mg\right)}{Surface\;area\;\left({dm}^{2}\right)\times Test\;duration\;\left(days\right)}$$

The concentration of released Fe was measured using an atomic absorption spectrometer (Varian AA-600) equipped with a dedicated graphite furnace, model GTA100, and Zeeman background correction. Both the field emission scanning electron microscope (FESEM) and the scanning electron microscope–energy dispersive X-ray spectrometer (SEM–EDX) were used to analyze the specimens before the sequential experiment began and again one month later.

The SEM analyses were performed at the Koptri Institute (Seoul, Republic of Korea). Analytical conditions included an accelerating voltage of 5 kV and a beam current of 30 nA for FESEM, while an accelerating voltage of 15 kV and a beam current of 50 nA were used for SEM–EDX.

## 4. Test Results

### 4.1. Effect of Coating Type on Measured Corrosion Rate and Fe Concentration

**Figure 7:**
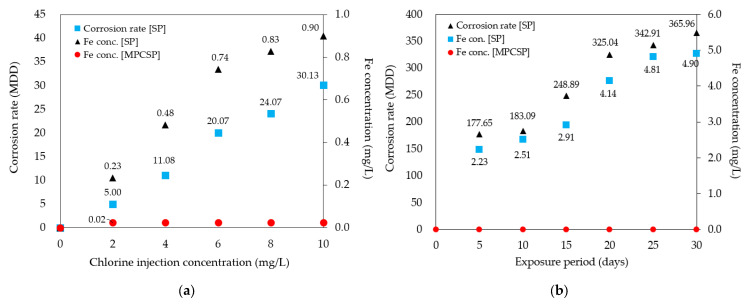
Effect of coating on measured corrosion rate and Fe concentration: (**a**) depends on chlorine injection concentration; (**b**) depends on exposure period (days).

The results of the batch leaching experiments confirmed that MPCSP exhibits superior corrosion resistance and significantly lower MDD compared to the control (SP) when exposed to chlorine, as shown in Figure 7a,b. Based on the results of the 7-day chlorine-induced corrosion test (see Figure 7a), the SP specimens showed greater iron leaching and higher corrosion rates. In contrast, the MPCSP specimens, with their internal polyethylene coating, are believed to have prevented iron leaching from the underlying carbon steel pipe, thereby preserving corrosion resistance against chlorine. Figure 7b shows the test results under the assumed extreme water pipe conditions. A 10 mg/L chlorine solution was injected, and the specimens were subjected to a 30-day continuous circulation experiment. The specimens were removed at 5-day intervals to measure the corrosion rate, based on weight loss, and the iron leaching concentration to evaluate the corrosion resistance to chlorine. The results confirmed that both the corrosion rate and iron leaching increased over time. Specifically, MPCSP specimens showed no significant change in weight and maintained an approximately zero-corrosion level, regardless of variations in chlorine injection concentration. No iron leaching was detected from the specimens, though iron concentrations measured at 0.02 mg/L likely originated from the tap water. Thus, it is expected that the MPP investigated does not react with chlorine at the concentrations introduced from water treatment plants. In contrast, SP displayed significant iron leaching and high corrosion rates under the same conditions. The batch leaching experiments suggest that the MPP coating inside SP prevents iron leaching from carbon steel pipes and maintains corrosion resistance against chlorine.

Similarly, sequential leaching experiments revealed that MPCSP specimens experienced no change in weight, maintained a zero-corrosion level, and showed no iron leaching for 30 days. Despite high concentrations of chlorine, the MPCSP exhibited no changes or surface corrosion, whereas the control suffered significant iron leaching and corrosion, leading to rust formation. Both the batch and sequential leaching experiments confirm that MPCSP possesses improved corrosion resistance.

**Figure 8:**
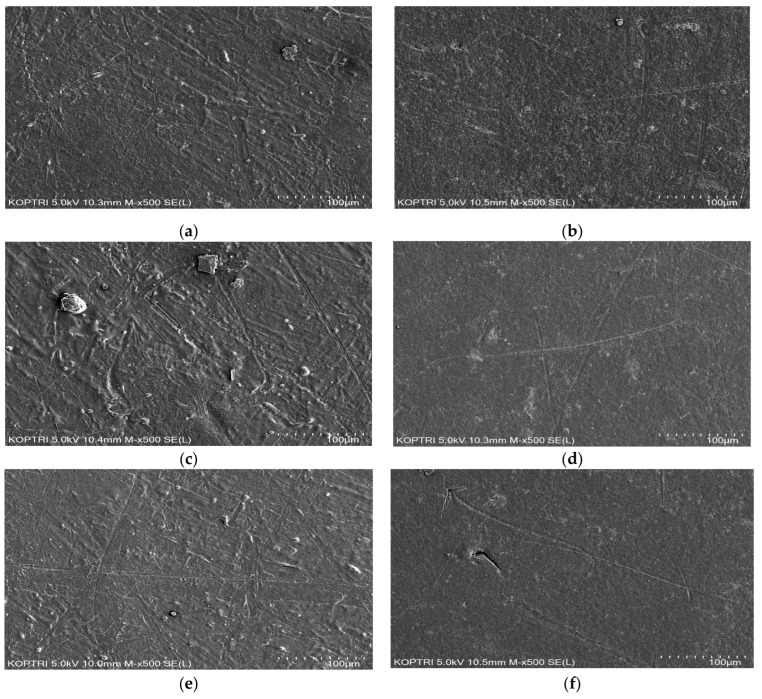
SEM results for some representative samples: (**a**) Point 1 without chlorine injection; (**b**) Point 1 with 1000 mg/L of chlorine injection; (**c**) Point 2 without chlorine injection; (**d**) Point 2 with 1000 mg/L of chlorine injection; (**e**) Point 3 without chlorine injection; (**f**) Point 3 with 1000 mg/L of chlorine injection.

The test results above can be attributed to the use of a thermal fusion method for lining the pipes with modified PE powder after special pretreatment. Additionally, the application of primer likely contributes to the strong adhesion of the coating material to the pipe, enhancing protection against corrosion. The synthetic resin PE coating prevented any lamination imperfections or defects on the coating surface during the experimental period, demonstrating low absorbency and improved corrosion resistance. Supporting this conclusion, the SEM results indicated no significant differences in surface corrosion between the MPCSP sample without chlorine injection and the sample after one month of injecting 1000 mg/L of chlorine. Both samples maintained uniform surfaces under magnification, as depicted in Figure 8b,d,f, with no detectable increase in porosity or surface roughness, confirming the absence of corrosion due to chlorine exposure. It should be noted that the three spots for each specimen, whether chlorine-injected or not, were randomly selected to investigate surface changes after the exposure test. In other words, the SEM images for Figure 8a,c,e and Figure 8b,d,f were taken from two separate samples, respectively.

As shown in Table 2, the SEM-EDX results confirmed that the primary components of MPCSP following the experiments were C and O, along with trace elements such as Na, Al, Cl, K, Ca, Ti, and Fe. The presence of chlorine might be attributed to the silicon treatment applied to the surface during sample preparation. One month after chlorine injection, elements other than C and O were detected at concentrations below 1% on average, indicating a minimal impact on corrosion. Based on previous research [30], the use of modified PE powder significantly influences the adhesion between the coating and the pipe, and resistance to water absorption can be improved through phosphate washing and chemical pretreatment. Moreover, three-layer PE coating improves the adhesion and chemical resistance of the pipe [31,32]. The corrosion prevention in these samples is largely due to the improved surface from chemical pretreatment and MPP application, alongside the superior corrosion resistance of the MPCSP test specimens. Overall, it can be concluded that the MPP coating effectively prevents the distribution of sediment and the attachment of limescale on the coating.

### 4.2. Effect of Coating Type on Blister Formation

**Figure 9:**
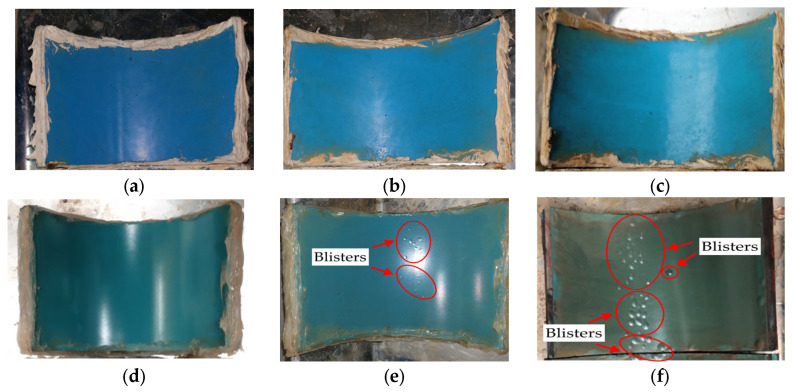
Blister formation on coating surface after wet immersion test: (**a**) 20 days (MPCSP); (**b**) 50 days (MPCSP); (**c**) 100 days (MPCSP); (**d**) 20 days (ECSP); (**e**) 50 days (ESCP); (**f**) 100 days (ESCP).

**Figure 10:**
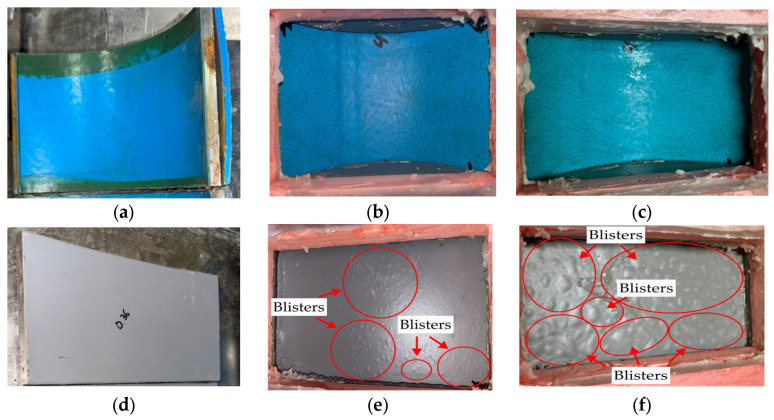
Blister formation on surface after thermal gradient test: (**a**) 0 day (MPCSP); (**b**) 20 days (MPCSP); (**c**) 100 days (MPCSP); (**d**) 0 day (ECSP); (**e**) 20 days (ECSP); (**f**) 100 days (ECSP).

Figure 9 displays the results of the wet immersion experiment on both MPCSP and ECSP specimens over a set period. Specifically, it presents test results from samples collected from MPCSPs (150A) and the same sizes of ECSP pipes. Samples from the MPCSPs showed no occurrence of blisters after 100 days. Conversely, specimens from ECSPs developed blisters after just 20 days, and by 100 days, most of the film was composed of blisters. As shown in Figure 10, the results from the temperature gradient test were quite similar to those from the wet immersion test. Namely, there was faster blister formation and propagation on the ECSP within the test period, as detected through visual inspection. The test results obtained in this study showed consistency with previous studies [32] that reported the improved mechanical strength and adhesion of three-layer polypropylene coatings applied to steel pipes.

Another reason for the reduced blister formation on MPCSPs compared to that on ECSPs might be attributed to the chemical pretreatment adopted in this study. However, further study considering more coating types and exposure conditions might be necessary.

## 5. Limitations and Future Studies

This study demonstrated the enhanced corrosion resistance of steel pipes coated with MPP, indicating the potential to extend their operational lifespan and reduce maintenance costs. This could be particularly beneficial for industries reliant on pipelines, such as water supply and oil transportation. However, the effectiveness of PE as a protective barrier depends on the quality of its adhesion to the steel pipes, a concern highlighted by Lee et al. [33], who found that poor bonding could significantly undermine corrosion resistance. Moreover, our study did not fully explore the material’s behavior under varying temperature ranges, raising concerns about its performance in high-temperature environments—a limitation also noted by Kim et al. [34]. The physical durability of PE, particularly its resistance to abrasion, remains uncertain and was not addressed in this study, which aligns with gaps identified in similar research [35]. Additionally, the scope of our research was limited to specific corrosive environments and did not assess the long-term performance of lined pipes, a concern echoed in studies by Singh et al. [36].

The corrosion resistance of surface-coated steel pipes is significantly influenced by the choice of coating materials and technologies [37,38,39,40,41,42,43]. Common coatings include epoxy, known for its strong adhesion and chemical resistance [44], and PE and PP, which are preferred for their moisture barrier properties. Ceramic coatings are also increasingly utilized for their durability and resistance to harsh conditions. Innovative application techniques such as plasma spraying and thermal spraying have greatly improved the uniformity and adhesion of these coatings. Despite these advancements, significant challenges remain in coating materials and technologies.

Further research is necessary to refine materials science and surface coatings to develop more resilient solutions and improve application technologies. Integrating sensors and monitoring technologies into coated pipes can facilitate early detection and mitigation of corrosion, enabling more effective maintenance strategies. Priority areas include developing new coating materials that withstand extreme environmental conditions—such as high temperatures, variable pH levels, and high salinity. Emerging materials like advanced polymer composites and ceramic-based coatings show promise for enhancing durability and reducing permeability. Additionally, innovative application techniques that ensure consistent coating thickness and improved substrate adhesion could significantly reduce the risks of blistering and subsequent corrosion. The environmental impact of current coating materials and processes also necessitates a continued focus on sustainability to minimize the ecological footprint of protective coatings.

This comprehensive approach advances corrosion science and supports industries reliant on these critical infrastructures, ensuring safer, more reliable, and cost-effective operations. This study contributes to an ongoing research project focused on developing smart pipe systems equipped with sensors for enhanced maintenance strategies. Further results from MPCSPs exposed to harsh marine environments will be reported in subsequent publications.

## 6. Conclusions

An experimental study on the chlorine-induced corrosion and blister formation on modified polyethylene-coated steel pipes (MPCSPs) was conducted with comparisons to conventional epoxy coated steel pipes (ECSPs). The following conclusions can be drawn:

1. MPCSPs demonstrated improved corrosion resistance compared to standard steel pipes (SPs), effectively preventing iron leaching and maintaining structural integrity under chlorine exposure.
2. The modified polyethylene coating exhibited robust resistance to environmental stressors such as moisture and temperature variations, ensuring stable performance without significant degradation.
3. MPCSPs did not develop blisters even after 100 days of immersion, whereas ECSPs began showing blisters as early as 50 days.
4. The three-layer coating system effectively prevented blister formation, outperforming conventional epoxy resin coatings.
5. The study highlights the potential of modified polyethylene to enhance both the chemical and physical properties of coatings, promoting more advanced and sustainable solutions.

Ongoing research into advanced coating technologies, including smart coatings with integrated sensors, is essential for enhancing predictive maintenance strategies and safety. Furthermore, improved coating technologies can significantly reduce both the economic costs and environmental risks associated with steel pipe corrosion. These findings suggest that further investigation into the long-term performance and environmental impact of innovative coating technologies is warranted.

## Figures and Tables

**Table 1:** Detailed sizes of specimens for blistering test (unit: mm).

Location	150A	600A	900A	1200A
a *	1.04	4.15	6.23	8.31
b *	24.88	99.54	149.30	199.08

* The location for a and b were specified in Figure 5.

**Table 2:** SEM-EDX results of MPCSP samples.

Elements	Unit	No Chlorine Injection	Chlorine Injection
C (Carbon)	wt %	75.925	89.225
O (Oxygen)		16.975	8.925
Na (Sodium)		0.275	0.17
Al (Aluminum)		0.075	0.085
Si (Silicon)		5.1	0.325
Cl (Chlorine)		0.1	0.17
K (Potassium)		0.15	0.1
Ca (Calcium)		0.125	0.13
Ti (Titanium)		0.6	0.5
Fe (Iron)		0.725	0.53
Total		100	100

## Data Availability

Data are contained within the article.
